# Qualitative exploration into reasons for delay in seeking medical help with diabetic foot problems

**DOI:** 10.1080/17482631.2021.1945206

**Published:** 2021-07-05

**Authors:** Michael Opeoluwa Ogunlana, Pragashnie Govender, Olufemi Oyeleye Oyewole, Adesola Christianah Odole, Jasola Love Falola, Olubiyi F. Adesina, Jabez Ariyo Akindipe

**Affiliations:** a Federal Medical Centre, Abeokuta, Nigeria; b College of Health Sciences, University of KwaZulu-Natal, Durban, South Africa; c Discipline of Occupational Therapy, School of Health Sciences, University of KwaZulu-Natal, Durban, South Africa; d Department of Physiotherapy , Olabisi Onabanjo University Teaching Hospital, Sagamu, Nigeria; e Department of Physiotherapy, University of Ibadan, Ibadan, Nigeria; f Department of Physiotherapy, Federal Medical Centre, Abeokuta, Nigeria; g Unit of Endocrinology, Department of Internal Medicine, Federal Medical Centre, Abeokuta, Nigeria; h Unit of Plastic Surgery, Department of Surgery, Federal Medical Centre, Abeokuta, Nigeria

**Keywords:** Diabetes, delay, foot, healthcare, amputation

## Abstract

**Purpose:**

Delay in reporting foot symptoms in patients with diabetes to health-care professionals is said to be responsible for limb amputation. While reasons for these delays have been investigated elsewhere, they are not well documented in Nigeria. This study explored the causes of delayed presentation in a Nigerian sample of patients with diabetic foot ulcers.

**Method:**

The study followed an explorative qualitative design in which the lived experience of eight participants with diabetes were explored. The participants completed in-depth interviews which were digitally audio-recorded and transcribed verbatim. Data were analysed thematically using deductive reasoning.

**Results:**

The study identified four themes which included knowledge and awareness of foot challenges, risk perception, health seeking triggers and behaviours and competing priority as the factors responsible for delay in presentation of diabetic foot complications.

**Conclusions:**

Limited knowledge and awareness and negative health seeking behaviours including self-management and consultation of traditionalists were the major reasons for delays.

## Introduction

The number of people who have diabetes in the world is growing day by day. In Sub-Saharan Africa, fast, uncontrolled urbanization and changes in standard of living is said to be responsible for the rising epidemic of diabetes mellitus and this observed increase presents a substantial public health and socioeconomic burden in the face of scarce resources (Mbanya et al., 2010). Diabetes challenges patients with numerous complications; even the proper treatment of Type 2 diabetes goes with cardiovascular diseases, neuropathy, nephropathy, retinopathy and diabetic foot syndrome (Anselmo et al., 2010). Foot ulcers occur in around four to 5% of patients with diabetes each year, and are associated with increased morbidity and mortality (Abdulghani et al., 2018; Cheer et al., 2009). In addition, more than 20% of patients, who suffer from diabetic foot syndrome, experience amputation during their lives (Pendsey, 2010). The need for limb amputation is reported to be associated with delay in reporting foot symptoms to health professionals (Van Battum et al., 2011). There is also a lack of consensus among healthcare professionals on the reliability of systemic indexes of inflammation and the risk to underestimate the severity of the foot impairment (Salutini et al., 2020).

Some researchers using quantitative approaches have examined the factors associated with delayed presentation of diabetic foot cases at regular hospitals elsewhere apart from Nigeria (Abbas, 2016; Jeffcoate & Harding, 2003) and surmised that unawareness of the presence of a foot ulcer, underestimating the significance of the problem and a lack of access to an appropriate health care professional are factors associated with delayed presentation of diabetic foot cases. Other studies that used qualitative approach have also suggested factors associated with delayed presentation of diabetic foot complications. Those factors include use of experimentation in health-seeking process and treatment strategies (Low et al., 2016a), switch between different alternative health care providers (Atwine et al., 2015), economic factors such as poverty and the high cost of biomedical care (Abdulrehman et al., 2016), a mixture of proper and improper information and beliefs (Sayampanathan et al., 2017) and delay in the health care process due to patients’ beliefs (Hjelm & Beebwa, 2013). Chithambo and Forbes conducted a qualitative study involving patients with diabetic foot syndrome in the UK. They concluded that ongoing foot care education is required to enhance patients’ knowledge on foot care, including the early warning signs of foot problems and what they should do to access help. It is also necessary to ensure that patients who have issues in being able to self-monitor their feet are subject to enhanced surveillance (Chithambo & Forbes, 2015).

The causes of delayed presentation in a Nigerian sample of patients presenting with diabetic foot may differ from that of a developed economy like the UK as the level of education are higher in the later. Therefore, exploring the causes of delay help-seeking in a Nigerian setting is imperative. Hence, this study explored the lived experience of individuals with diabetic foot complication and provides greater insight into the causes of delay in reporting foot problem from patients’ perspective.

## Methods and materials

The relevant domains of the consolidated criteria for reporting qualitative research (COREQ) (Booth et al., 2014) are used to report the methods in this study. The study followed an explorative qualitative design in order to explore and describe the lived experience of participants with diabetes. The participants selected for this study were defined as delayed health care seekers based on the following selection criteria (i) Patients that required intravenous antibiotics on their first presentation to the diabetic foot clinic, (ii) Patients that presented with gangrenous ulcers, (iii) Patients who may require either partial or full amputation of the lower limbs at presentation. Patients with a recorded diagnosis of severe depression and psychotic disorders were excluded. Participants that fulfilled the inclusion criteria were recruited via the diabetes foot clinic of a tertiary hospital in Nigeria. A reasonably homogenous purposive sample (Patton, 2014) of eight participants were recruited with completion of eight individual interviews. The one participant was excluded due to an accident that resulted in a traumatic amputation. Following ethical approval from the Health Research Committee of a tertiary hospital in, Nigeria and signed informed consent, participants were interviewed. The authors designed a semi-structured interview (see Appendix) with open-ended questions after extensive literature review, discussions, and pilot testing. This interview format was used by one of the authors (MOO) during data collection. This author occupied a hybrid position of a researcher and a medical rehabilitation specialist. His pre-conceived opinion of the need for early intervention was moderated by the interview format that allowed open-ended dialogue from the research participants. Peer debriefing (Patton, 2014) was done during analysis to limit the potential biases associated with the positionality of the interviewing author. Demographic data were analysed descriptively. The interview data were digitally audio-recorded and transcribed verbatim. Authors employed deductive reasoning in thematically analysing the data (Braun et al., 2019). Analysis of each individual interview occurred, followed by pattern matching across cases (Miles et al., 2013). This was then transformed into a narrative account supported by verbatim extracts from each participant. Authors reviewed and audited the themes to ensure that they appeared to be grounded in the transcripts and well represented within the data with adequate examples thereby increasing the trustworthiness of the study. An audit trail also assisted in reducing bias in the study (Patton, 2014).

## Results

The lived experiences of eight participants that were diagnosed with diabetes mellitus and who had presented to the study site for a scheduled amputation are described. The demographic profile of these participants is illustrated in Table I followed by a description of the four emergent themes in this study.

**Table I.** Participant demographics (n = 8)

Pseudonym	*Sunday*	*Adubi*	*Segun*	*Peter*	*Sarah*	*Adijat*	*Muji*
***Age***	55 years	73 years	37 years	51 years	62 years	65 years	59 years
***Gender***	Male	Female	Male	Male	Female	Female	Female
***Marital Status***	Monogamous marriage	Polygamous marriage	Monogamous Marriage	Monogamous Marriage	Monogamous marriage	Polygamous marriage	Polygamous marriage
***Educational Level***	Secondary	No formal Education	Tertiary	Tertiary	No formal education	No formal education	No formal education
***Religion***	Christianity	Christianity	Islam	Christianity	Christianity	Islam	Islam
***Employment Status***	Retired	Trader	Teacher	Company Worker	Trader	Trader	Petty Trader
***Diagnosis of Diabetes***	2 years	5 years	10 years	17 years	2 months	1 year	2 months
***Presenting Complaint***	Boil on the leg	Foot Ulcer	Foot Ulcer	Foot Ulcer	Foot Ulcer	Foot Ulcer	Foot ulcer
***Presentation to Hospital***	28 weeks after onset	4 weeks after onset	6 weeks after onset	16 weeks after onset	12 weeks after onset	104 weeks after onset	4 weeks after onset
***Scheduled amputation***	2 skin grafts	Below knee amputation	Below knee amputation	Above knee amputation	Below knee amputation	Above knee amputation	Below knee amputation

### Knowledge and awareness of foot challenges

Participants in this study generally described having limited knowledge and awareness of diabetic foot care. When probed directly on information received on foot care, the majority indicated having not received this information.

*“I was not told, but I observed that in the middle of the night, when I urinate like four times, I believe something is wrong” (Sunday)*

*“I don’t know anything about it” (Adijat)*

*“I just know when there is pain … ” (Adubi)*

One participant however indicated that he had been briefed on relevant precautions.

*“I used to go to the clinic, and I was told a lot about taking care of my leg and was given some precautions” (Segun)*

Notwithstanding this, in their description of presenting complaints prior to hospitalization, it appeared as though participants were sensitized with diabetic education and information from various sources, including medical personnel.

*“One nurse told me not to scratch it.” “ … When the leg (ulcer) burst(ed), she told me to go to hospital (Segun)*

*“I was told by a girl living with me to take care of my leg” (Adubi)*

*“I was briefed when I attended the diabetic centre … ” (Peter)*

### Risk perceptions

Two of the participants presented with insight into the aetiology and potential risks.

*“It might clot because of the diabetes nature. I also know as a diabetes patient, it’s easy for infection to go into the leg and cause other damages” (Peter)*

*“I’m diabetic and my sugar level is not controlled. Nothing more” (Segun)*

The remaining participants, however, appeared not to have intellectual insight into their condition.

*“ … when one is above 55 years, the body starts misbehaving. That’s my belief because I did not inherit it from anybody.” (Sunday)*

*“I hit my leg against something, and I did not know it would lead to something serious” (Muji)*

### Health seeking triggers and behaviours

Despite having received this information from various sources, the timeliness of presentation to the hospital, for interventions appeared to be influenced by the participant’s notions and application of self-management processes including traditional care.

*“ … before going to the clinic, I treated it locally by placing a knife inside the fire with palm oil and I dropped the hot oil on the entrance of the nail on my foot” (Segun)*

*“I bought antibiotics and managed it by myself for three months” (Sarah)*

*“I started using herbal medicine, but other complications set in.” (Peter)*

Moreover, non-adherence to prior diabetic education was noted.

*“I did not take to all instructions given to me” (Segun)*

*“I took care of it … ” “I applied soap and leaf … ” (Adijat)*

### Competing priorities

A number of competing priorities emerged on the part of patients and healthcare settings which included the patient’s competing priorities (a lack of finances and the presence of comorbidities) as well as competing priorities of the healthcare setting (delayed interventions and delayed secondary referrals).

The major competing priority on the part of patients was finance. Sarah described that lack of finances impeded her ability to access the relevant care timeously. She expressed,

*“ … it was me not having money that made me to be like this.” (Sarah)*

Adijat voiced her co-morbidity of a cardiovascular accident (CVA) as possibly causing her foot ulcer.

*“I noticed the sore on my leg gradually … ” I think the stroke (that) caused it” (Adijat)*

Delayed or sub-optimal intervention by healthcare providers was another sub-theme that emerged as a competing priority on the part of healthcare settings. Sunday describes how an auxiliary nurse at a local clinic had initially managed him and delayed the appropriate interventions required,

“ *… she cleaned and bandaged it, but she discovered she could not handle it anymore and told me to go to the hospital.” (Sunday)*

Peter described how postponement of his appointments led to spread of infection further compromising his condition. He explained,

*“I was told only the right toe has been affected and that the only alternative is to cut off the right toe immediately. But they didn’t cut it because it was not their clinic day and they kept postponing … so the whole thing degenerated, so I had (have) to cut from the level of the leg.” (Peter)*

## Discussion

This study has highlighted some reasons as to why individuals who were living with diabetes and reporting foot complications, presented with delays in seeking medical help. A strong theme that emerged included limited knowledge and awareness around diabetic foot complications. Majority of the participants in this study lack knowledge about diabetes, especially with respect to foot complications and are limited in their understanding of how to care for their feet as individuals living with diabetes. Generally, people living with diabetes in sub-Saharan Africa have been shown to have limited knowledge and awareness around diabetic complications (Abdulrehman et al., 2016; Chiwanga & Njelekela, 2015; Goie & Naidoo, 2016; Hjelm & Beebwa, 2013; Hjelm & Mufunda, 2010; Kassahun et al., 2016; Mufunda et al., 2012; Ntontolo et al., 2017; Ugwu et al., 2019; Zimmermann et al., 2018). Despite this, it appears as those who are highly educated among the participants in this study, presented with fair knowledge and awareness, albeit suboptimal, based on current diabetes foot care clinical practice guidelines (Pérez-Panero et al., 2019). Suboptimal knowledge about diabetes foot complications is not limited to people with diabetes from Africa, as studies from other parts of the world suggest the existence of limited knowledge amongst people living with diabetes (Chithambo & Forbes, 2015; Perera et al., 2013; Rouyard et al., 2017). This calls for more appropriate and effective diabetes care education for those who have been diagnosed with the condition. Quality information and health literacy should characterize the knowledge offered to them. This will reduce the effect of any negative information they might have received from the multiple sources as noted from this study (Low et al., 2016b). A well-developed educational intervention for diabetes care have shown to improve patients’ knowledge, awareness, self-care and health promotion behaviours (Chahardah-Cherik et al., 2018; Mohammad & Khresheh, 2018).

Evidence of low-risk perception of diabetic foot complications was suggested in a systematic review (Rouyard et al., 2017). In this study, we observed low-risk perception of diabetes as a factor for delays in seeking medical help for diabetic foot complications, which concurs with a previous study by Chithambo and Forbes (2015). Two of the participants, who appeared to have some form of risk perception, were those who have a tertiary education in this study. This was aligned with a previous study that postulated higher levels of education increases risk perception among people with diabetes (Lamchahab et al., 2011). Again, this call for well-tailored diabetes foot care education to encourage health promotion behaviour and early presentation of diabetic foot complications.

Another theme that emerged relating to delayed presentation of diabetic foot complications is health-seeking triggers and behaviours. It would appear that the pre-conceived notions our participants had inevitably influenced their health-seeking behaviours concerning diabetic foot complications. Many of the participants have consulted non-biomedical methods and self-managed themselves prior to presenting at a health facility. In sub-Saharan Africa, the hybridity of diabetes care is common. Persons living with diabetes consult traditional healers, self-medicate, and combine advice from various healers. This is in addition to accessing care from biomedical healthcare professionals and auctioning their own religious beliefs, which contributed to this treatment discourse (Abdulrehman et al., 2016; Alavi et al., 2011; Atwine et al., 2015; Zimmermann et al., 2018). Despite self-detection of diabetic foot complications early among the participants in this study, they did not present early to a health facility. This may be as a result of the participants’ perceived severity of their ulcer presentation as insignificant. It has been suggested that perceived seriousness of diabetic ulcer presentation influence health-seeking behaviours while those who perceived the diabetes ulcer as trivial delayed seeking biomedical care (Chithambo & Forbes, 2015). It is observed among participants in this study that cultural practices/beliefs override prior education when seeking medical help for diabetic foot complications. This is in agreement with a study from Kenya where socio-cultural factors influence self-management of diabetic foot complications (Abdulrehman et al., 2016). Moreover, it was noted that a lack of adherence to prior diabetes foot care education was responsible for delays in seeking help for some of the participants who had been attending a clinic prior to the development of the diabetic foot complications. Again, reinforcing health literacy that promotes healthy behaviours is required for people living with diabetes so as to reduce complications and enhance adherence.

An additional theme that emerged in this study is the competing priorities that were responsible for delays in the presentation of diabetic foot complications. A lack of finance was one of the major constraints in presentation of diabetic foot complications among the participants in this study. Previous studies have reported the narrative relating to the impediments people living with diabetes face in managing their foot complications as the high cost of treatment (Zimmermann et al., 2018) and thus, preventing them from reporting the difficulties early. In Nigeria, health insurance is just evolving and many patients pay out of pocket for their treatment. Therefore, disruption in economic circumstances is a major problem among persons living with diabetes in Nigeria as the cost may be too high to afford. This may as well explain their negative health-seeking behaviours or health inaction around diabetic foot complications (Abdulrehman et al., 2016). This corroborated Mufunda et al.’s study which reported prolonged disruption in economic circumstances among people living with diabetes demonstrating a conflict between willingness and ability to comply thereby leading to health inaction (Mufunda et al., 2012).

This study also revealed that some of the delays were related to delayed secondary referral and delayed intervention. This is worrisome as those complications that could have been prevented or minimized were exacerbated by the health professional not responding timeously as noted by a previous study (Chithambo & Forbes, 2015). Improving patients-health professionals’ ratio may minimize delayed interventions as well as developing professional education on prompt detection of diabetic foot complications and early referral.

The aforementioned factors responsible for delayed presentation of diabetic foot complications add to the existing burden of diabetic-ulcers in Nigeria (Ugwu et al., 2019). This burden and delayed presentation of foot complications may be minimized or may be reversed through thorough foot care education, community enlightenment programmes and establishment of well-trained diabetic foot care teams (Ugwu et al., 2019). We, however, acknowledge the need for further exploration of the reasons for delay among patients with diabetic-ulcers from other regions of Nigeria.

This study has some clinical implications. The importance of diabetic foot care education cannot be over-emphasized. Effort should be more directed at individuals living with diabetes and their relations to prevent diabetic foot complications. Such educational programs should include risk assessment and awareness, prompt attention to foot and proper health-seeking behaviours. Since some of the participants seek help from non-biomedical sources, educational effort in diabetic foot care should also be directed to these non-biomedical sources. The outcomes of this study also call for affordable foot health care services for individuals living with diabetes in Nigeria. The government should possibly find a way of encouraging more Nigerians to enrol in national health insurance to facilitate prompt and quality coverage.

## Conclusion

This study sought to explore and understand the lived experiences of participants who were diagnosed with diabetic foot complications and who were considered delayed health care seekers. The emergent themes such as limited knowledge and awareness of foot challenges, risk perception, health-seeking triggers and behaviours and competing priority were highlighted as the reasons for delayed presentation of patients with diabetic foot challenges. Diabetes targeted enlightenment programmes, that covers both preventive and promotive care are required in order to ensure early referral and early presentation at health care facilities. Diabetic foot care teams are needed to prevent the high incidence of diabetic-related amputations. Inclusion of culturally sensitive and appropriate material to negotiate myths and traditional beliefs in line with western medicine may be necessary in the Nigerian population. This may ensure adherence to available diabetic management regimen by the patients.

## Appendix.

**SCHEDULE OF INTERVIEW**

Preambles

- Welcome the subject
- Re-explain concept of research/interview
- Answer patients’ questions about interview

Proceed to Interview

- Please kindly tell me about how this foot challenge started?

- How much information do you have on foot care? (Prompt—Elaborate please on available information)
- Where and how did you get the information on foot care? (Prompt—Did you think the information was applicable to you?)

- Tell me if you can do an independent or supervised foot inspection
- When did you become aware of the foot problem?
- How did it start? (Onset)
- What did you do when you observed the foot problem (Action taken)

- What did you think caused this foot problem (Aetiological perception)

- What was done for self-care
- Did you ignore the foot problem initially, why did you ignore it?
- Why did you go to the hospital? (Motivations to present at hospital)
- Do you have other health challenges (Influence or effects of other illness)
- Did you have previous unhelpful hospital visits? Was there delayed in secondary referral?

Thank you for participating in our research.
